# The impact of dietary inflammation index on benign prostatic hyperplasia: insights from patient data and animal models

**DOI:** 10.3389/fnut.2026.1760675

**Published:** 2026-02-24

**Authors:** Jingwei Ke, Sheng Wang, Xinyang Liao, Youliang Qian, Hai Tang, Xing Liu

**Affiliations:** 1 Department of Urology, The Affiliated Traditional Chinese Medicine Hospital, Southwest Medical University, Luzhou, Sichuan, China; 2 The Department of Urology, West China Hospital, Department of Sichuan University, Chengdu, Sichuan, China; 3 Department of Urology, West China School of Medicine, Sichuan University, Sichuan University Affiliated Chengdu Second People's Hospital, Chengdu Second People's Hospital, Chengdu, China

**Keywords:** animal models, benign prostatic hyperplasia, clinical research (CRE), inflammation, Mendelian randomization

## Abstract

**Background:**

Benign prostatic hyperplasia (BPH) is a common chronic condition among elderly males, typically manifesting as lower urinary tract symptoms (LUTS), including increased urinary frequency, urgency, nocturia, urinary stream splitting, and dysuria. Previous reports have indicated a potential association between dietary habits and BPH; however, the specific causal relationship between dietary factors and prostatic hyperplasia remains unclear. Therefore, this study aimed to investigate the potential causal relationship between the dietary inflammation index (DII) and BPH through a cross-sectional cohort analysis, two-sample Mendelian randomization (TS-MR), and complementary animal experiments.

**Methods:**

DII and BPH were defined using data from the National Health and Nutrition Examination Survey (NHANES), and their association was investigated. We then used TS-MR to screen nine dietary preferences and evaluate their causal effects on BPH risk. To validate these findings, we conducted external dietary interventions on rats according to three dietary patterns (baseline diet group, pro-inflammatory diet group, and anti-inflammatory diet group) to modulate dietary preferences, and assessed prostatic hyperplasia as well as systemic and local inflammation in the rats using H&E, Masson, and IHC staining, and ELISA assays.

**Results:**

Higher DII scores were significantly associated with increased BPH risk (fully adjusted OR = 1.07, 95% CI: 1.03–1.12, *P* < 0.001), with a primarily linear dose–response relationship. MR analysis revealed that genetically predicted anti-inflammatory diet was inversely associated with BPH risk (OR = 0.80, 95% CI: 0.66–0.98, *P* = 0.034), providing genetic evidence of causality. *In vivo*, rats on a pro-inflammatory diet exhibited a significantly elevated prostate index, pronounced epithelial hyperplasia, and increased collagen deposition, along with higher serum levels of IL-6, TNF-α, and IL-1β. Conversely, anti-inflammatory diets mitigated these effects, preserving normal glandular architecture and reducing inflammatory marker expression. Collectively, these findings demonstrate that pro-inflammatory dietary patterns promote benign prostatic enlargement and inflammation both systemically and locally.

**Conclusion:**

Our integrated population-based, genetic, and experimental evidence supports a causal role of dietary inflammatory load in the development of BPH. Chronic consumption of pro-inflammatory diets may promote BPH through sustained systemic and prostate-specific inflammation, while anti-inflammatory dietary patterns may confer protective effects. These findings highlight the potential of dietary modulation as a preventive and therapeutic strategy for BPH management.

## 1 Introduction

The prostate is a walnut-sized gland situated inferior to the male bladder and surrounding the urethra. Its core functions include the secretion of prostatic fluid, a major component of semen, and the action of its muscular tissue, which assists in the regulation of urination and contracts during ejaculation to facilitate the closure of the bladder neck and the propulsion of seminal fluid. Benign prostatic hyperplasia (BPH) is a prevalent chronic condition in middle-aged and elderly males, characterized by prostate enlargement that can lead to bladder, urinary tract, or kidney-related symptoms, such as partial or complete urethral obstruction. It is one of the most frequently diagnosed diseases in the clinical practice of urology and andrology (1).

In 2021, BPH imposed a substantial global disease burden, with incident cases reaching 137.88^*^10^5^ (95% UI 109.08–170.15), representing a 115.23% increase compared with 1990. In the same year, the global prevalence of BPH rose to 1125.02^*^10^5^ cases (95% UI 881.32–1426.34), corresponding to an age-standardized prevalence rate of 2,782.59 per 100,000 persons (95% UI, 2,191.58–3,508.04), underscoring the growing and considerable global health burden of this condition (2, 3). Previous studies have indicated that the histological condition of BPH tends to persistently rise with advancing age (4), and the increasing trend in the number of BPH patients is more pronounced in emerging aging nations such as China. However, to date, the academic community has yet to fully elucidate the specific pathogenesis of BPH. Potential underlying risk factors may include metabolic syndrome, diabetes mellitus, obesity, hypertension, dietary habits, and levels of sex hormones (1).

Chronic inflammation is primarily characterized by the sustained elevation of inflammatory levels over an extended period and is significantly influenced by dietary factors (5). Previous research has established that chronic inflammation plays a pivotal role in the pathogenesis of age-related diseases (6, 7). BPH is a classic age-related chronic inflammatory disease. As a long-term, slow-acting factor influencing the human body, diet can lead individuals to gradually form pro-inflammatory or anti-inflammatory dietary patterns based on their preferences. Consequently, dietary patterns may differentially influence systemic inflammatory responses, underscoring the importance of elucidating the role of dietary factors in the pathogenesis of BPH (8–10). Lifestyle modifications, particularly dietary interventions such as the Mediterranean diet, have been shown to reduce the incidence of BPH in the population, highlighting its potential as a management strategy for BPH (11, 12).

Dietary factors influence chronic disease risk largely through their effects on oxidative stress and energy balance, which are closely interconnected determinants of metabolic health (13–15). Excessive intake of energy-dense, pro-inflammatory foods promotes reactive oxygen species (ROS) production and disrupts antioxidant defenses, leading to oxidative damage to cellular macromolecules and impairment of metabolic signaling pathways, thereby contributing to the development of cardiometabolic and other chronic diseases (15, 16). At the same time, sustained positive energy balance driven by unhealthy dietary patterns facilitates adiposity and insulin resistance, exacerbates low-grade chronic inflammation, and further amplifies oxidative stress, creating a self-perpetuating pathogenic cycle (17). In contrast, adherence to nutrient-dense, anti-inflammatory dietary patterns rich in fiber, unsaturated fats, and bioactive compounds supports redox homeostasis and metabolic efficiency, helping to maintain energy balance and reduce chronic disease susceptibility (18). Dietary inflammation index (DII) is a novel tool that has emerged in recent years to explore the inflammatory contributions of various dietary components (19, 20). Although previous studies have suggested a potential association between dietary factors and the onset and progression of BPH, the conclusions drawn from these studies remain to be further substantiated, owing to limitations such as sample size or research design. To address this gap, we utilized data from the National Health and Nutrition Examination Survey (NHANES) to investigate the relationship between the DII and BPH. Furthermore, we conducted two multi-omics Mendelian randomization (MR) studies to examine the impact of different dietary preferences on BPH. In addition, we employed an animal model using rats to investigate the effects of different dietary patterns on prostatic hyperplasia.

## 2 Materials and methods

This study encompasses three primary components. Initially, we utilized dietary and BPH data from the NHANES database to define the DII and to assess the prevalence of BPH among participants. Through methods such as multivariate logistic regression, linear trend analysis, non-linear testing, and subgroup analysis, we explored the relationship between the DII and BPH. Subsequently, we extracted Instrumental variables (IVs) from Genome-wide association studies (GWAS) related to dietary preferences. Employing a two-sample MR approach, we evaluated the impact of different dietary preferences on susceptibility to BPH. Finally, we conducted an external intervention on the dietary preferences of rats by grouping them into three dietary patterns (reference diet group, pro-inflammatory diet group, and anti-inflammatory diet group). We then assessed the occurrence of prostatic hyperplasia in rats through methods such as hematoxylin and eosin (HE) staining, Masson's trichrome, Picro-Sirius Red Stain, immunohistochemistry (IHC), and Enzyme-linked immunosorbent assay (ELISA), thereby exploring the causal relationship between human dietary preferences and BPH.

### 2.1 Cross-sectional cohort study

#### 2.1.1 Study population

**Figure 1:**
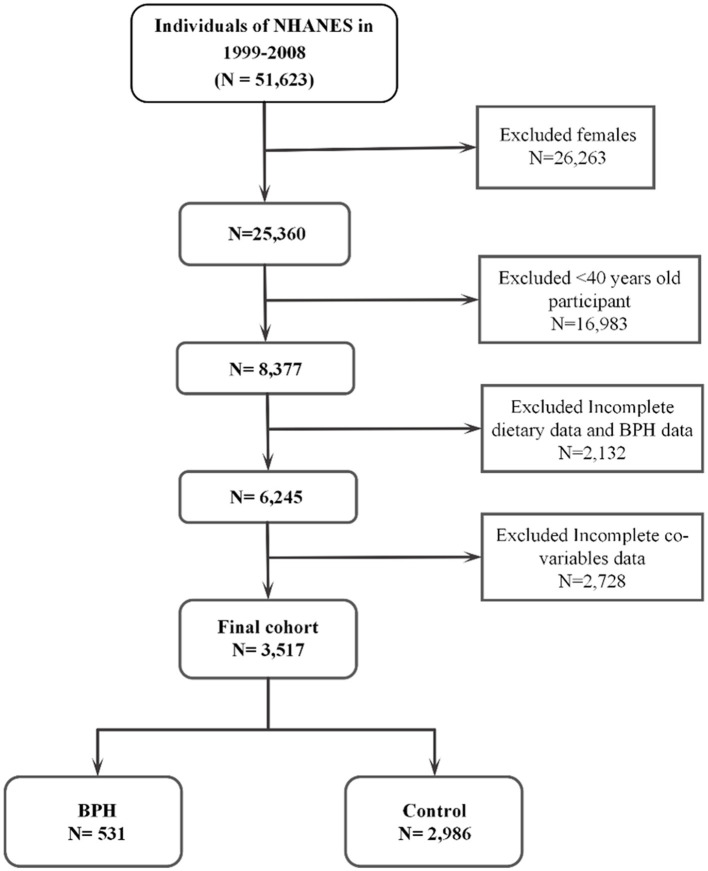
Flow chart of the selection process for participants in the NHANES 1999–2008.

NHANES is a nationally representative cross-sectional survey targeting the non-institutionalized civilian population of the United States, which is designed as a national program aimed at assessing the health and nutritional status of the American people (21). The NHANES protocol was approved by the Institutional Review Board (IRB) of the National Center for Health Statistics (NCHS), and informed consent was obtained from all participants. The project is conducted biennially and boasts a sample size of over 8,000 individuals. To investigate the relationship between DII and BPH, this study utilized data from five cycles of the NHANES spanning from 1999 to 2008. The selected cycles were chosen due to their comprehensive coverage of variables required for dietary and BPH data, with all data meticulously processed in strict accordance with standardized protocols. Our analysis adhered strictly to pre-established inclusion and exclusion criteria, which included female participants, individuals under the age of 40, and those lacking complete dietary, prostate, or covariate information. Initially, our participant pool consisted of 51,623 individuals. However, after applying these stringent exclusion criteria, our study ultimately encompassed 3,517 participants (Figure 1).

#### 2.1.2 Assessment of dietary inflammatory potential

The dietary inflammatory potential of habitual dietary intake was assessed using the DII, a literature-derived scoring system that integrates the pro- and anti-inflammatory effects of individual dietary components (20, 22). The index is constructed from evidence linking specific foods and nutrients to circulating inflammatory biomarkers, including interleukins, tumor necrosis factor-α, and *C*-reactive protein, thereby enabling a standardized evaluation of diet-related inflammatory burden across populations (20). In large-scale epidemiological studies such as NHANES, DII scores are calculated using dietary data obtained from 24-h dietary recall interviews, which capture detailed information on daily food and beverage consumption (23). Individual nutrient intakes are standardized against a global reference database, converted into centered percentile scores to minimize skewness, and subsequently weighted by their respective inflammatory effect scores derived from the literature (20). The weighted scores of all available dietary components are then summed to generate an overall DII score, with higher values indicating a more pro-inflammatory dietary pattern (24). Notably, the DII has demonstrated robust validity across diverse populations and remains reliably computable even when fewer than the full set of food parameters are available, supporting its applicability in large observational datasets and chronic disease research (25, 26). Ultimately, the sum of the results obtained from all dietary components yields the total Dietary Intake Index value for the study subjects. Detailed information regarding the dietary parameters used in the study is provided in the Supplementary Table S1.

#### 2.1.3 Assessment of BPH

Regarding the diagnosis of BPH, male participants were queried regarding their prostate health with the following questions: “Have you ever been told by a doctor or health professional that you have any disease of the prostate? This includes an enlarged prostate.” If the response was affirmative, an additional question was posed: “Have you been told you have prostate enlargement? Is it benign enlargement? ”

Only participants who answered affirmatively to both questions were categorized as having BPH. Participants with missing data or those diagnosed with malignant hyperplasia were excluded from the study (27–29). These questions are represented by the NHANES questionnaire codes KIQ490, KIQ121, and KIQ141. Participants with other prostate conditions, non-benign enlargement, or missing data were excluded.

#### 2.1.4 Covariates

Covariates were selected *a priori* based on biological plausibility and previous literature regarding BPH and metabolic health. Demographic and lifestyle variables included age, race/ethnicity, sex, smoking status, alcohol consumption, educational attainment, household income, and poverty-to-income ratio. Clinical and metabolic factors included body mass index (BMI), diabetes status, and Metabolic syndrome (MetS). MetS was defined according to established criteria and incorporated as a composite metabolic risk indicator to account for its recognized association with BPH (30). All covariates were derived from standardized NHANES questionnaires, physical examinations, and laboratory assessments.

### 2.2 Mendelian randomization

#### 2.2.1 GWAS data sources

We identified genetic tools for BPH from the latest R12 GWAS summary data in the Finngen database, which encompasses a total of 194,710 European individuals, including 41,137 patients with BPH. The Finngen database is an integrated project that combines the digital health records from the Finnish Health Registry with the genetic data from the Finnish Biobank (https://www.finngen.fi/en). Diagnoses of BPH patients were made according to the ICD-10 diagnostic criteria.

The GWAS data related to inflammatory dietary phenotypes were sourced from the GWAS Catalog. To specifically investigate the impact of inflammatory-related dietary preferences on the risk of BPH, we have selectively filtered and identified nine dietary preference GWAS. These datasets encompass information from up to 421,155 participants registered in the UK Biobank, and detailed information regarding the GWAS summary statistics used in this study can be found in the Supplementary Table S2.

#### 2.2.2 Genetic instruments selection criteria

All genetic instruments (SNPs) associated with inflammatory dietary intake were selected at the genome-wide significance level (*P* < 5 × 10^−8^). Using the 1,000 Genomes Project reference panel based on European ancestry, we performed linkage disequilibrium (LD) clumping to identify independent SNPs for each trait. The LD threshold was set at *r*^2^ < 0.001 within a clumping window of 10 kb. To ensure valid causal inference in the MR analyses, the effects of SNPs on exposure and outcome were harmonized so that they corresponded to the same effect allele. We conducted this harmonization using the “TwoSampleMR” R package and excluded palindromic SNPs with ambiguous strand orientation. To minimize potential confounding, SNPs that were strongly associated with the outcome (*P* < 5 × 10^−5^) were removed prior to analysis. The Steiger directionality test was performed for each instrumental SNP to confirm that the causal direction was from exposure to outcome rather than the reverse. In addition, we calculated the *F*-statistic for each instrument to assess its strength, with values below 10 indicating a weak instrument (Supplementary Table S3).

#### 2.2.3 Mendelian randomization statistical analysis

The random-effects Inverse-variance weighted (IVW) method was used as the primary analytical approach for all MR analyses. The IVW model provides a weighted regression of SNP-specific causal estimates and yields robust causal inferences, even in the presence of balanced horizontal pleiotropy (31).

To validate the core assumptions of the univariable MR analysis, we performed several sensitivity analyses, including the weighted median, MR-Egger regression, Bayesian weighted Mendelian randomization (BWMR), and MR Pleiotropy RESidual Sum and Outlier (MR-PRESSO) methods. The weighted median estimator computes the weighted median of individual SNP-specific causal effects, providing a consistent estimate when more than 50% of the total weight comes from valid instrumental variables (32). The MR-Egger regression allows for non-zero average horizontal pleiotropic effects but at the cost of reduced statistical power. The BWMR approach, based on a variational expectation-maximization (VEM) algorithm, applies Bayesian weighting to mitigate violations of instrumental variable assumptions caused by pleiotropy, thereby enabling causal inference even under pleiotropic conditions (33). The MR-PRESSO distortion test detects significant differences between causal estimates before and after the removal of outlier SNPs (34). We further applied the Egger intercept test to evaluate potential directional pleiotropy and performed the Steiger directionality test to confirm the causal direction between exposure and outcome. Cochran's *Q* test was used to assess heterogeneity among instrumental SNPs and to evaluate the consistency of MR assumptions across analyses. Based on these MR estimations and sensitivity analyses, we considered a causal inference to be robust and credible when the following criteria were met: the causal estimates from the four MR approaches and sensitivity analyses showed consistent directions of effect; the MR-Egger intercept indicated no evidence of pleiotropy.

All the above processes were carried out in R 4.1.0 (https://www.R-project.org/). R package “TwoSampleMR” [The MR-Base platform supports systematic causal inference across the human phenome]. And “MRPRESSO” [The MR-Base platform supports systematic causal inference across the human phenome] were used to perform MR and sensitivity analyses.

### 2.3 Animal experiments

#### 2.3.1 Animal model

Twenty-four specific pathogen-free (SPF) male Sprague-Dawley (SD) rats (6 weeks old, body weight 200 ± 20 g) were procured from Beijing Vital River Laboratory Animal Technology Co., Ltd. (Beijing, China). All animals were housed at the Experimental Animal Center of West China Hospital, Sichuan University (Chengdu, Sichuan, China), four rats per cage, with *ad libitum* access to food and water. The housing environment was maintained under controlled conditions (temperature: 23 °C ± 2 °C; humidity: 55% ± 5%; 12-h light/12-h dark cycle). Rats were maintained under sterilized conditions, with cages subjected to ultraviolet or high-pressure steam sterilization. Upon arrival, animals were randomly assigned to three dietary groups (*n* = 8 per group). The control group received a standard pellet diet (3.02 kcal/g; 24% protein, 62% carbohydrate, 13% fat), which served as a nutritionally balanced and inflammation-neutral reference diet. To model a pro-inflammatory dietary pattern, rats were fed a high-fat, high-sucrose diet (4.87 kcal/g; 16.4% protein, 41.1% carbohydrate, 42.5% fat), a composition that reflects a positive DII profile and has been widely used to induce chronic low-grade systemic inflammation in rodent models (20, 22, 35). The anti-inflammatory diet was formulated by modifying the AIN-93 purified diet to enrich ω-3 polyunsaturated fatty acids and dietary fiber (3.22 kcal/g; 24.8% protein, 58.4% carbohydrate, 16.8% fat), thereby representing a negative DII profile (36). This design was based on evidence that marine-derived ω-3 polyunsaturated fatty acids suppress pro-inflammatory mediator production and modulate immune signaling pathways, while high dietary fiber enhances short-chain fatty acid production and reduces pro-inflammatory cytokine expression in animal models (37–39). This graded dietary intervention strategy, grounded in the DII framework, was designed to enable a translational evaluation of the impact of dietary inflammatory load on prostatic hyperplasia. All animals were maintained on their respective diets for 12 weeks. At the end of the experimental period, rats were anesthetized by isoflurane inhalation (induction at 3%−4% and maintenance at 1.5%−2.0%). Adequate depth of anesthesia was confirmed prior to sample collection. Blood and prostate tissues were harvested under deep anesthesia to minimize pain and distress, and animals were subsequently euthanized by cervical dislocation in accordance with established guidelines for laboratory animal welfare. Serum samples were centrifuged and stored at −80 °C, while prostate tissues were weighed and either fixed in 4% paraformaldehyde or snap-frozen in liquid nitrogen for further analysis. All animal experimental protocols were reviewed and approved by the Animal Ethics Committee of West China Hospital, Sichuan University, to ensure adherence to the principles of Replacement, Reduction, and Refinement (3Rs) and to promote animal welfare and scientific rigor in experimental design. Approval was granted on 3 September 2024 (approval no. 20240903006). All animal procedures were conducted in strict accordance with the National Institutes of Health (NIH) Guide for the Care and Use of Laboratory Animals.

#### 2.3.2 Enzyme-linked immunosorbent assay (ELISA)

Serum concentrations of TNF-α, IL-1β, and IL-6 were quantified using commercial ELISA kits (Bioswamp, Wuhan, China) in strict accordance with the provided instructions. Absorbance was measured at 450 nm with a microplate reader. Standard curves, generated from the known concentrations of the provided standards, were used to interpolate the cytokine concentrations in the rat samples. Details of the raw measurement data for ELISA experiments, including absorbance OD values at 450 nm and standard curves, are provided in Supplementary Table S4.

#### 2.3.3 H&E staining and immunohistochemical staining (IHC)

Tissue samples were fixed in 4% paraformaldehyde, embedded in paraffin, and sectioned at a thickness of 4 μm. The sections were subjected to hematoxylin and eosin (HE) staining for histopathological evaluation, and Masson's trichrome staining to visualize collagen deposition. All stained sections were examined under an optical microscope (Olympus, Tokyo, Japan). The collagen-positive areas in Masson's trichrome-stained sections and the Picro-Sirius Red-positive areas in Picro-Sirius Red stain were quantified using ImageJ software.

For IHC analysis, sections were deparaffinized, rehydrated, and incubated overnight at 4 °C with specific primary antibodies. After treatment with appropriate secondary antibodies, antigen visualization was achieved using diaminobenzidine (DAB), followed by counterstaining with Mayer's hematoxylin. Immunoreactivity was evaluated with the IHC Profiler plugin in ImageJ: National Institutes of Health, Bethesda, MD, United States (40), which integrates staining intensity (average gray value) and the percentage of positive area to assign a composite score: high positive (4), positive (3), low positive (2), or negative (1). Details regarding primary antibodies, including sources and dilution ratios, are provided in Supplementary Table S5.

#### 2.3.4 Statistical analysis

Results are expressed as mean ± standard deviation (SD) from a minimum of three independent replicates. Group comparisons were conducted using Student's *t*-test (for two groups) or one-way ANOVA (for multiple groups). All statistical analyses and graph generation were carried out with GraphPad Prism version 9.0: GraphPad Software, San Diego, CA, United States. A *P*-value of less than 0.05 was deemed statistically significant, with the following markers indicating degree of significance: ^*^P < 0.05, ^**^P < 0.01, ^***^P < 0.001, and ^****^P < 0.0001.

## 3 Results

### 3.1 Cross-sectional study

#### 3.1.1 Baseline characteristics

A total of 3,517 participants were included in the analysis, of whom 531 were classified as having BPH and 2,986 served as controls. The mean age was 69 years in the BPH group and 54 years in the non-BPH group. Survey-weighted baseline characteristics stratified by BPH status are presented in Table 1. Compared with participants without BPH, individuals with BPH were more likely to be older, non-Hispanic White, and to have higher educational attainment and Poverty-to-income ratio (PIR). In addition, the BPH group exhibited a higher prevalence of cardiometabolic comorbidities, including hypertension, coronary heart disease, and MetS. Lifestyle-related differences were also observed, with a greater proportion of current or former smokers among participants with BPH. Collectively, these findings indicate that BPH was associated with both adverse metabolic profiles and distinct sociodemographic characteristics in this population.

**Table 1:** Characteristics of participants classified according to BPH.

**Characteristics**	**Total (*N* = 3,517)**	**BPH**		***p*-value**	**method**
		**No (*****N*** = **2,986)**	**Yes (*****N*** = **531)**		
DII, mean ± sd	−2.86 ± 3.87	−2.93 ± 3.89	−2.51 ± 3.36	1.06E−02	Welch *t*-test
Age, median (IQR)	56 (47, 67)	54 (46, 65)	69 (60, 76)	4.89E−80	Wilcoxon
**Race**, ***n*** **(%)**					
Non-Hispanic black	571 (16.2%)	509 (17%)	62 (11.7%)	7.63E−12	Chisq test
Non-Hispanic white	2,052 (58.3%)	1,669 (55.9%)	383 (72.1%)		
Mexican American	656 (18.7%)	605 (20.3%)	51 (9.6%)		
Other race—including multi-racial	73 (2.1%)	66 (2.2%)	7 (1.3%)		
Other hispanic	165 (4.7%)	137 (4.6%)	28 (5.3%)		
**Education level**, ***n*** **(%)**					
Less than 9th grade	468 (13.3%)	422 (14.1%)	46 (8.7%)	5.00E−04	Fisher test
9–11th grade (includes 12th grade with no diploma)	464 (13.2%)	423 (14.1%)	41 (7.7%)		
High school grad/GED or equivalent	800 (22.7%)	697 (23.3%)	103 (19.4%)		
College graduate or above	907 (25.8%)	714 (23.9%)	193 (36.3%)		
Some college or AA degree	878 (25%)	730 (24.4%)	148 (27.9%)		
**Marital status**, ***n*** **(%)**					
Married	2,491 (70.8%)	2,086 (69.9%)	405 (76.3%)	4.94E−09	Chisq test
Divorced	362 (10.3%)	324 (10.9%)	38 (7.2%)		
Widowed	177 (5%)	129 (4.3%)	48 (9%)		
Never married	216 (6.1%)	200 (6.7%)	16 (3%)		
Living with partner	180 (5.1%)	162 (5.4%)	18 (3.4%)		
Separated	91 (2.6%)	85 (2.8%)	6 (1.1%)		
PIR, median (IQR)	3.18 (1.60, 5)	3.07 (1.5, 5)	3.65 (2.05, 5)	3.98E−07	Wilcoxon
BMI, median (IQR)	27.9 (25.2, 31.1)	27.9 (25.2, 31.0)	28.2 (25.3, 31.3)	5.14E−01	Wilcoxon
**CHD**, ***n*** **(%)**					
No	2,977 (84.6%)	2,579 (86.4%)	398 (75%)	1.77E−11	Chisq test
Yes	540 (15.4%)	407 (13.6%)	133 (25%)		
**Diabetes**, ***n*** **(%)**					
No	3,061 (87%)	2,613 (87.5%)	448 (84.4%)	9.98E−02	Chisq test
Borderline	70 (2%)	55 (1.8%)	15 (2.8%)		
Yes	386 (11%)	318 (10.6%)	68 (12.8%)		
**Drinking**, ***n*** **(%)**					
Non or light	3,084 (87.7%)	2,587 (86.6%)	497 (93.6%)	6.88E−06	Chisq test
Heavy	433 (12.3%)	399 (13.4%)	34 (6.4%)		
**Hypertension**, ***n*** **(%)**					
No	2,164 (61.5%)	1,896 (63.5%)	268 (50.5%)	1.31E−08	Chisq test
Yes	1,353 (38.5%)	1,090 (36.5%)	263 (49.5%)		
**Smoking**, ***n*** **(%)**					
Never smoker	1,211 (34.4%)	1,065 (35.7%)	146 (27.5%)	1.01E−03	Chisq test
Former smoker	569 (16.2%)	469 (15.7%)	100 (18.8%)		
Current smoker	1,737 (49.4%)	1,452 (48.6%)	285 (53.7%)		
UA, median (IQR)	6 (5.2, 6.9)	6 (5.2, 6.9)	6 (5.2, 7)	4.84E−01	Wilcoxon
**MetS**, ***n*** **(%)**					
No	2,816 (80.1%)	2,410 (80.7%)	406 (76.5%)	2.39E−02	Chisq test
Yes	701 (19.9%)	576 (19.3%)	125 (23.5%)		

BPH, Benign prostatic hyperplasia; IQR, Interquartile range; PIR, Poverty-income ratio; DII, Dietary inflammation index; BMI, Body mass index; CHD, Coronary heart disease; UA, Urinary acid; MetS, Metabolic syndrome.

#### 3.1.2 The association between DII and BPH

Our analyses demonstrated a consistent association between the DII and BPH across multiple regression models (Table 2). In survey-weighted logistic regression analyses, higher DII scores were associated with an increased risk of BPH in both unadjusted and adjusted models. In the univariate model, each one-unit increase in DII was associated with a higher odds of BPH. This association remained statistically significant after sequential adjustment for demographic factors, including age, race/ethnicity, and body mass index, as well as additional sociodemographic and clinical covariates. Notably, in the fully adjusted model, higher DII scores continued to show a robust positive association with BPH risk, indicating that the observed relationship was independent of multiple potential confounders.

**Table 2:** Weighted multivariable logistic regression analysis of the association between DII and BPH risk.

**DII score**	**Detail**	**BPH**	
		**OR (95% CI)**	***P***
Model I^*^	DII	1.09 (1.05, 1.13)	2.78E−05
Model II	DII + age + BMI + race	1.06 (1.02, 1.11)	7.28E−03
Model III	DII + age + BMI + race + educational level + marital status + PIR	1.07 (1.03, 1.12)	3.48E−03
Model IV	DII + age + BMI + race + educational level + marital status + PIR + smoking + drink	1.07 (1.03, 1.12)	3.04E−03
Model V	DII + age + BMI + race + educational level + marital status + PIR + smoking + drink + diabetes + hypertension + CHD + UA + MetS	1.07 (1.03, 1.12)	2.86E−03

^*^Model I: unadjusted (crude association), Model II: adjusted for demographic factors (age, BMI, race), Model III: further adjusted for socioeconomic status (educational level, marital status, PIR), Model IV: additionally adjusted for behavioral factors (smoking status, alcohol consumption), Model V: fully adjusted for all preceding variables plus clinical comorbidities (diabetes, CHD, UA and MetS).

BPH, Benign prostatic hyperplasia; PIR, Poverty-income ratio; DII, Dietary inflammation index; BMI, Body mass index; CHD, Coronary heart disease; UA, Urinary acid; MetS, Metabolic syndrome; OR, Odds ratio.

To further characterize the nature of this association, we examined potential nonlinear relationships between DII and BPH risk (Table 3). The linear term of DII was significantly associated with BPH, whereas the quadratic term was not statistically significant. These findings suggest that the association between DII and BPH risk is predominantly linear, with no evidence supporting a nonlinear dose-response relationship. Collectively, these results indicate that increasing dietary inflammatory potential is linearly and consistently associated with a higher risk of BPH.

**Table 3:** Nonlinear associations of DII with BPH risk before and after adjustment.

**Variable**	**Estimate**	**SE**	***t*-value**	***P*-value**
**Unadjusted**				
DII	0.10190793	0.025825112	3.946079	1.81E−04
I (DII^2^)	0.002227304	0.001365703	1.630885	1.07E−01
**Adjusted**				
DII	0.095923723	0.02817478	3.4045952	1.33E−03
I (DII^2^)	0.002894197	0.001324021	1.859146	7.36E−02

#### 3.1.3 Subgroup analyses

**Figure 2:**
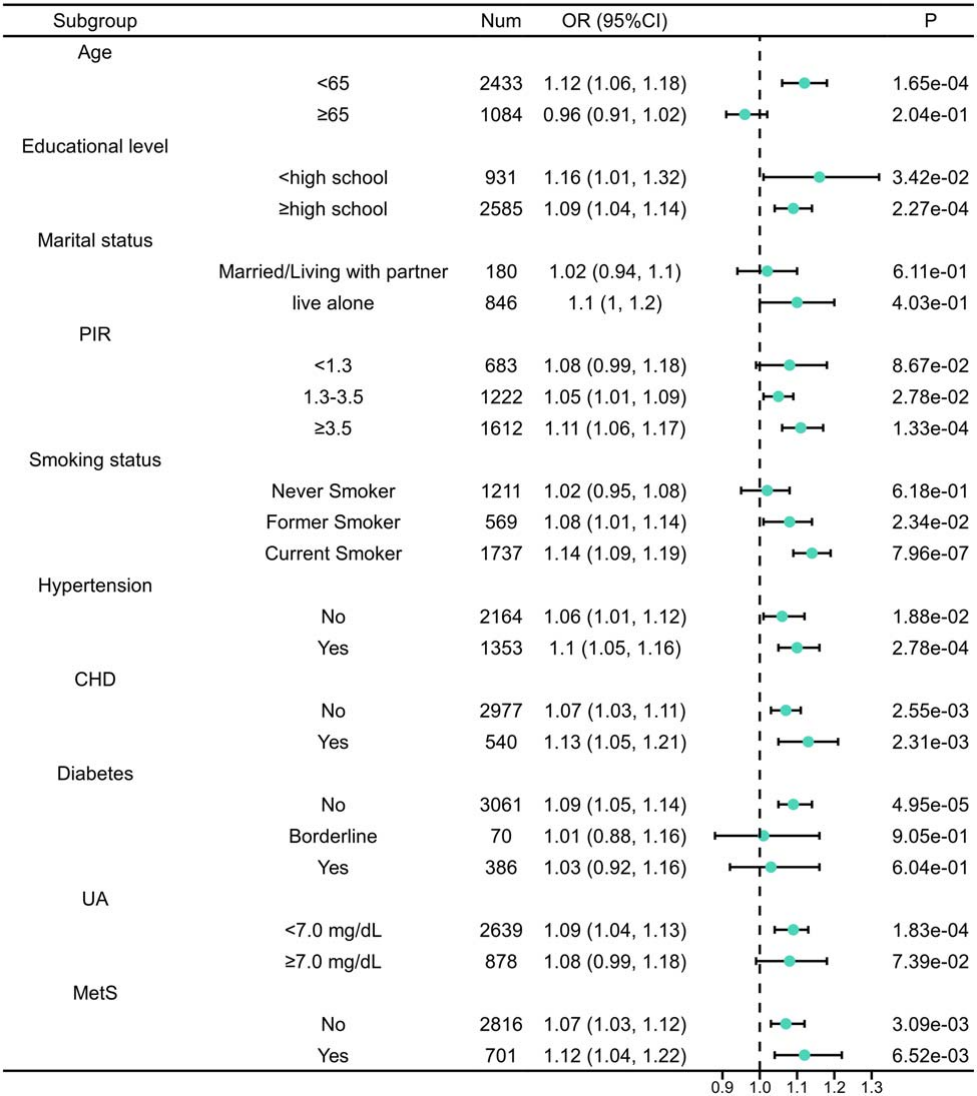
Subgroup analysis for the association between DII and BPH. PIR, Poverty-income ratio; DII, Dietary inflammation index; CHD, Coronary heart disease; UA, Uric acid; MetS, Metabolic syndrome.

Subgroup analyses demonstrated that the positive association between the Dietary Inflammation Index (DII) and BPH risk was generally consistent across a wide range of demographic, socioeconomic, lifestyle, and clinical characteristics (Figure 2). The association appeared more pronounced among participants younger than 65 years compared with those aged 65 years or older. Comparable positive associations were observed across categories of educational attainment, marital status, and income level. With respect to lifestyle and clinical factors, higher DII scores were associated with increased BPH risk in both smokers and non-smokers, as well as in participants with and without hypertension or coronary heart disease. When stratified by metabolic status, the positive association between DII and BPH remained evident in both participants with and without MetS, with no substantial attenuation of effect size across strata. Similarly, the association persisted across categories of serum uric acid levels, with a slightly stronger association observed among individuals with hyperuricemia. Although the association was attenuated and did not reach statistical significance in participants with established diabetes, it remained significant in non-diabetic and pre-diabetic individuals. Overall, these subgroup analyses indicate that the association between dietary inflammatory potential and BPH risk is robust across most subgroups, with only modest variation in magnitude.

### 3.2 Mendelian randomization

The details of the instrumental variables (IVs) for each exposure are provided in Supplementary Table S2. All selected single-nucleotide polymorphisms (SNPs) passed Steiger filtering (*P* < 0.05), confirming the correct directionality from the exposure to the outcome. The strength of the instruments was robust, with F-statistics ranging from 29.76 to 425.93 (all >10), exceeding the conventional threshold and indicating minimal weak instrument bias (41).

**Figure 3:**
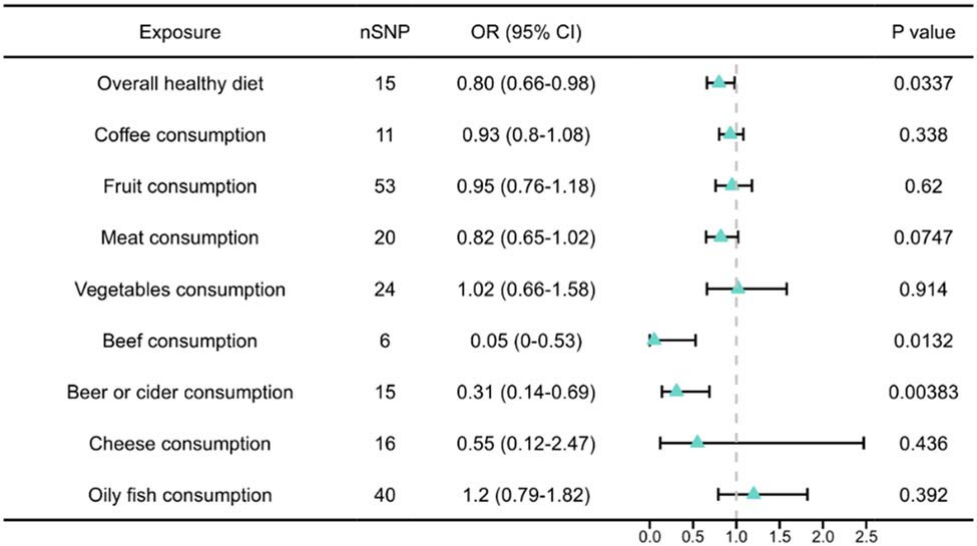
MR-IVW analyses results of genetically predicted dietary traits with risk of BPH. MR, Mendelian randomization; IVW, Inverse variance weighted method.

TS-MR analyses were performed to assess the potential causal effects of genetically predicted dietary patterns and specific food intakes on BPH risk. A genetically predicted overall healthy diet was significantly associated with a reduced risk of BPH (IVW: OR = 0.80, 95% CI: 0.66–0.98, *P* = 0.034). Among specific food items, genetically predicted higher beef consumption (IVW: OR = 0.05, 95% CI: 0.00–0.53, *P* = 0.013) and beer or cider consumption (IVW: OR = 0.31, 95% CI: 0.14–0.69, *P* = 0.004) were also inversely associated with BPH risk. In contrast, no statistically significant associations were observed for genetically predicted coffee intake, fruit and vegetable consumption, cheese intake, or oily fish consumption (all *P* > 0.05). A borderline inverse association was noted for meat consumption (IVW: OR = 0.82, 95% CI: 0.65–1.02, *P* = 0.075). Detailed information is presented in Figure 3.

To assess the robustness of the results, we performed sensitivity analyses. The direction of causal estimates was consistent across all positive findings. Steiger filtering confirmed no reverse causality bias in all analyses (Supplementary Table S6). Although a few outliers were detected by MR-PRESSO, the results remained consistent after their removal. Furthermore, the MR-Egger intercept test indicated no significant horizontal pleiotropy. These detailed results are presented in Supplementary Table S7.

In summary, the MR results offer genetic support for a causal relationship in which a healthier, anti-inflammatory diet reduces BPH risk. These findings genetically establish a causal connection between pro-inflammatory dietary patterns and an elevated risk of BPH.

### 3.3 Vivo experiments on rats

#### 3.3.1 Dietary inflammatory load increases prostate index in rats

After 12 weeks of dietary intervention, rats receiving the pro-inflammatory diet exhibited a significant increase in body weight–adjusted prostate mass compared with both the control and anti-inflammatory diet groups (*P* < 0.01). The prostate index (PI) was highest in the pro-inflammatory group (mean ± SD: 0.810 ± 0.054 mg/g), moderate in the control group (0.679 ± 0.041 mg/g), and lowest in the anti-inflammatory diet group (0.585 ± 0.044 mg/g), indicating that high dietary inflammatory load promotes prostate enlargement (Table 4).

**Table 4:** Effects of the diet type on body weight, prostatic wet weight, and the prostatic index.

**Groups**	**Body weight**	**Prostatic wet weigh**	**Prostatic index**
	**(g)**	**(mg)**	**(mg/g)**
Control	462.36 ± 12.7	313.92 ± 16.7	0.679 ± 0.041
Pro-inflammatory diet	498.35 ± 21.2^**^	403.65 ± 20^**^	0.810 ± 0.054^**^
Anti-inflammatory diet	447.18 ± 25.4^**^	261.43 ± 12.6^**^	0.585 ± 0.044^**^

Data are expressed as the mean ± SD.

^**^Indicates *P* < 0.01 compared with the control group.

#### 3.3.2 Histopathological alterations of the prostate

**Figure 4:**
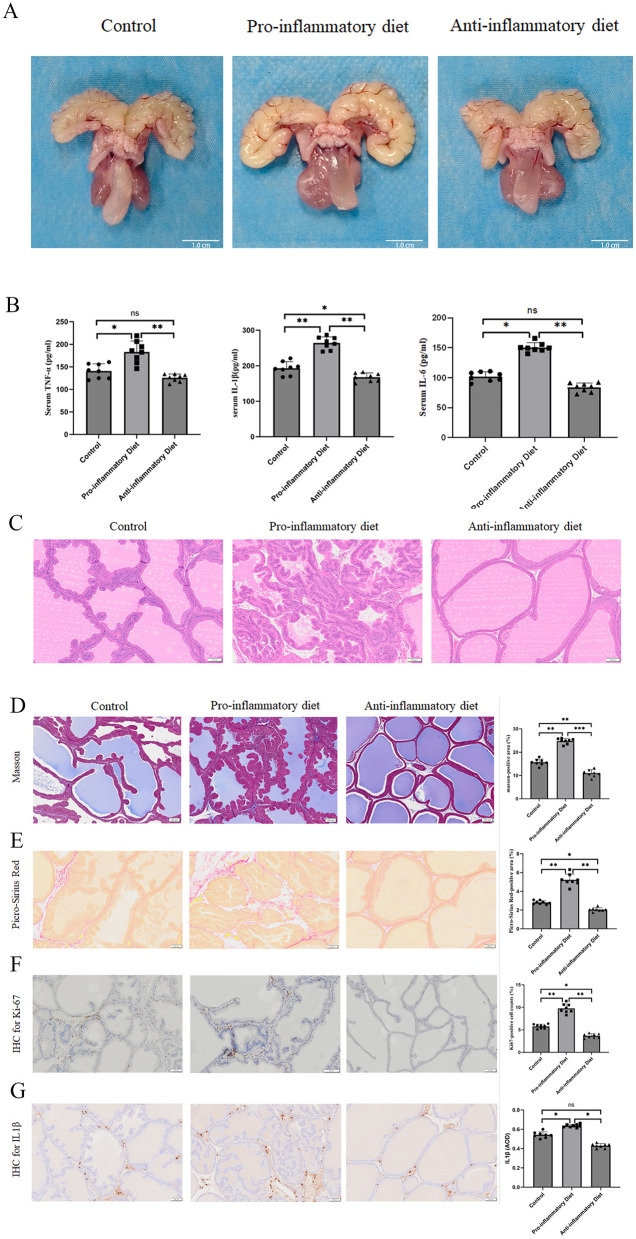
Impact of different dietary patterns on the prostate *in vivo*. **(A)** Representative gross images of prostates from the control, pro-inflammatory diet, and anti-inflammatory diet groups. Images were processed using ImageJ software (NIH, Bethesda, MD, United States) to ensure standardized cropping and scale bar calibration. **(B)** Bar plots showing the serum levels of TNF-α, IL-1β, and IL-6 in control, pro-inflammatory diet, and anti-inflammatory diet groups. **(C)** Representative HE staining of prostate samples in control, pro-inflammatory diet, and anti-inflammatory diet groups. **(D)** Representative masson staining of prostate samples from the control, pro-inflammatory diet, and anti-inflammatory diet groups. **(E)** Representative Picro-sirius Red staining of prostate samples from the control, pro-inflammatory diet, and anti-inflammatory diet groups. **(F)** Representative IHC staining of Ki-67 in prostate samples from the control, pro-inflammatory diet, and anti-inflammatory diet groups. **(G)** Representative IHC staining of IL1e in prostate samples from the control, pro-inflammatory diet, and anti-inflammatory diet groups. Data are expressed as the means ± SEMs (*p < 0.05, **p < 0.01, ***p < 0.001, ns, not significant).

Hematoxylin–eosin (H&E) staining (Figure 4C) revealed typical hyperplastic features in the pro-inflammatory diet group, including multilayered epithelial proliferation, papillary infoldings into glandular lumina, and reduced luminal area. The control prostates showed regular acinar morphology with a single epithelial layer, whereas the anti-inflammatory diet group preserved normal architecture with minimal epithelial thickening.

#### 3.3.3 Collagen deposition and stromal remodeling

Masson's trichrome staining (Figure 4D) demonstrated abundant collagen deposition and pronounced stromal fibrosis in the pro-inflammatory diet group, while the anti-inflammatory group showed sparse collagen fibers comparable to controls. Similarly, Picro-Sirius Red staining under polarized light (Figure 4E) confirmed increased total collagen content and thick, red-stained collagen bundles in the pro-inflammatory group, whereas thinner, green-stained fibers predominated in the anti-inflammatory group, indicating reduced fibrosis.

#### 3.3.4 Cell proliferation and local inflammation

Immunohistochemical staining for Ki-67 (Figure 4F) demonstrated a marked elevation in proliferative activity within the epithelial cells of the pro-inflammatory group, consistent with hyperplastic histology. In contrast, Ki-67 positivity was sparse in the anti-inflammatory diet group, suggesting inhibition of epithelial proliferation. Furthermore, IHC for IL-1β (Figure 4G) showed intense cytoplasmic staining in both glandular epithelium and stromal regions of the pro-inflammatory group, while weak or minimal IL-1β expression was detected in the anti-inflammatory and control groups. These findings highlight the dietary modulation of local inflammatory signaling within the prostate.

#### 3.3.5 Systemic inflammatory profile

Consistent with the histological observations, serum ELISA assays revealed significantly higher levels of IL-6, TNF-α, and IL-1β in the pro-inflammatory diet group compared with the other groups (*P* < 0.05). The anti-inflammatory diet group exhibited the lowest cytokine concentrations, approaching baseline values of the controls.

#### 3.3.6 Integrated interpretation

Collectively, these data demonstrate that chronic intake of a pro-inflammatory diet induces BPH–like changes characterized by epithelial proliferation, stromal fibrosis, and both local and systemic inflammation. Conversely, an anti-inflammatory diet attenuates these pathological alterations, preserving normal glandular morphology and suppressing inflammatory cytokine expression.

These findings provide experimental evidence supporting the causal association inferred from NHANES and MR analyses, indicating that inflammatory dietary patterns contribute to BPH pathogenesis through activation of systemic and local inflammatory pathways (Figures 4A–G).

## 4 Discussion

In this study, we provide converging epidemiological, genetic, and experimental evidence supporting a causal role of dietary inflammatory load in the development of BPH. By integrating population-based data from NHANES, two-sample Mendelian randomization analyses, and controlled dietary intervention experiments in rats, we demonstrate that pro-inflammatory dietary patterns, quantified by a higher DII, are consistently associated with an increased risk of BPH, whereas anti-inflammatory dietary patterns confer protective effects. Importantly, the concordance of observational associations, genetically predicted causal estimates, and *in vivo* pathological changes strengthens the robustness of our findings and reduces the likelihood that the observed associations are driven by residual confounding or reverse causation. Together, these results position dietary inflammation as a previously underappreciated but potentially modifiable contributor to the pathogenesis of BPH.

The epidemiological evidence from NHANES revealed that higher DII scores were positively associated with BPH prevalence, consistent with earlier reports associating inflammatory diets with chronic diseases such as metabolic syndrome, cardiovascular disease, and certain cancers (42–44). This aligns with growing recognition that “inflammaging”—the chronic, low-grade inflammation that accompanies aging-plays a pivotal role in the onset and progression of BPH (45, 46). MR findings extend this evidence by providing causal inference, showing that genetically determined predispositions toward inflammatory dietary patterns confer higher risk of BPH. Building on these findings, MR analyses provided genetic evidence supporting a causal relationship, demonstrating that genetically determined predispositions toward pro-inflammatory dietary patterns are associated with increased BPH risk. Together, these genetic results reinforce the notion that dietary inflammation is not merely a correlate of metabolic dysfunction but may act as a direct driver of prostate pathology (47–49).

Given the well-established link between BPH and metabolic syndrome, we further explored whether metabolic status modified the observed association between dietary inflammatory load and BPH risk (30). Subgroup analyses stratified by MetS status demonstrated that higher DII scores were associated with increased BPH risk in both individuals with and without MetS, with comparable effect estimates across strata. These findings suggest that dietary inflammation may contribute to prostatic hyperplasia through mechanisms that are not entirely dependent on overt metabolic syndrome. Rather than acting solely as a downstream manifestation of metabolic dysregulation, dietary inflammatory burden may represent an upstream or parallel pathway linking diet to prostate pathology.

Notably, moderate heterogeneity was observed in the MR analyses, reflecting variability in causal estimates across individual genetic instruments. Such heterogeneity is not unexpected in MR studies of complex dietary exposures, as dietary traits encompass heterogeneous behavioral patterns and may influence disease risk through multiple biological pathways. Importantly, the direction of effect remained consistent across complementary MR methods, and sensitivity analyses did not indicate substantial horizontal pleiotropy. Taken together, these findings suggest that the observed heterogeneity likely reflects underlying biological complexity rather than violations of core MR assumptions, thereby supporting the robustness of the inferred causal relationship. In this context, the causal evidence provided by MR further strengthens the epidemiological observations by minimizing confounding related to lifestyle factors and reverse causation.

Our results highlight systemic inflammation as the bridge between diet and prostatic hyperplasia. Pro-inflammatory diets, typically high in refined carbohydrates, saturated fats, and processed meats, stimulate circulating cytokines and oxidative stress markers, thereby activating nuclear factor κB (NF-κB) and Janus kinase/signal transducer and activator of transcription (JAK/STAT) pathways (50–52). These signaling cascades upregulate growth factors such as transforming growth factor-β (TGF-β) and fibroblast growth factor-2 (FGF2), which promote fibroblast proliferation and extracellular matrix deposition in the prostate (53, 54). Our animal data corroborate these findings, showing increased collagen accumulation and inflammatory infiltration in rats fed a high-DII diet. Conversely, anti-inflammatory dietary patterns—characterized by higher intake of fruits, vegetables, omega-3 fatty acids, and polyphenols—are known to suppress these inflammatory mediators (55, 56). These findings support the biological plausibility of dietary inflammation as a modifiable determinant of prostate tissue remodeling.

A particularly innovative aspect of this study lies in the proposal and experimental support of the gut–prostate axis as a mechanistic pathway linking diet, inflammation, and prostate pathology. Mounting evidence suggests that gut dysbiosis plays a critical role in systemic inflammation and metabolic diseases (57–59). High-DII diets induce shifts in gut microbial composition, reducing beneficial short-chain fatty acid (SCFA)–producing taxa such as Faecalibacterium and Bifidobacterium, while enriching pathobionts such as Escherichia–Shigella and Enterobacteriaceae (60–62). These alterations compromise intestinal barrier integrity, facilitating translocation of microbial products such as lipopolysaccharides (LPS) into circulation. Circulating LPS activates Toll-like receptor 4 (TLR4) and downstream NF-κB signaling within the prostate, resulting in local cytokine production, oxidative stress, and cellular proliferation (63–65). This process mirrors mechanisms described in metabolic liver disease and obesity, further supporting the systemic impact of gut-derived inflammatory mediators (66, 67). Our animal model, in which pro-inflammatory diets elevated both systemic and prostatic inflammatory markers, provides experimental validation for this hypothesis.

Beyond inflammation, the gut–prostate axis may exert its influence through endocrine and metabolic interactions. The gut microbiota modulates androgen metabolism by producing enzymes involved in deconjugating and transforming steroid hormones (68, 69). Dysbiosis may therefore alter local androgenic signaling within the prostate, contributing to tissue proliferation and hormonal imbalance. In parallel, microbial metabolites such as Short-chain fatty acids (SCFAs) can regulate histone deacetylase activity, influencing gene expression related to inflammation and cellular proliferation (70). The intersection of microbial metabolism, hormonal signaling, and immune activation underscores the complexity of gut–prostate communication, suggesting that dietary modulation of the microbiome may represent an upstream intervention point for BPH management.

The interplay between diet, inflammation, and the gut microbiota also raises the possibility of personalized nutrition strategies in BPH prevention. The Mediterranean and Dietary Approaches to Stop Hypertension (DASH) diets, both characterized by low DII scores, have been associated with reduced lower urinary tract symptoms (LUTS) and improved metabolic profiles in men with BPH (12, 71). Similarly, probiotic and prebiotic interventions have shown potential in restoring gut microbial balance and attenuating systemic inflammation in metabolic disorders (72, 73). Incorporating such dietary approaches into clinical practice could provide synergistic benefits alongside pharmacologic therapies targeting androgen or smooth muscle pathways. The DII, as a composite metric reflecting inflammatory dietary exposure, may thus serve not only as a research tool but also as a practical biomarker for individualized risk assessment and dietary counseling.

Several limitations warrant consideration. First, although MR strengthens causal inference, it cannot fully capture nonlinear effects or time-dependent dietary exposures. Second, while the animal model provides mechanistic insight, extrapolation to human disease should be made with caution. Finally, detailed characterization of gut microbiota and downstream immune signaling pathways was beyond the scope of the present study and merits further investigation. Addressing these aspects in future work may help refine the mechanistic links between diet, inflammation, and prostatic pathology.

In summary, our findings extend the current understanding of BPH by highlighting dietary inflammatory load as a relevant and potentially modifiable contributor to disease development. Traditionally regarded as primarily driven by aging and hormonal factors, BPH may also be shaped by long-term dietary exposures that influence systemic and local inflammatory states. From a methodological perspective, the integration of observational epidemiology, genetic causal inference, and experimental validation provides a robust framework for studying diet–disease relationships. Collectively, these results suggest that consideration of dietary inflammatory potential may offer complementary insights into the prevention and management of BPH.

## 5 Conclusion

The study provides convergent epidemiological, genetic, and experimental evidence that pro-inflammatory dietary patterns contribute causally to the development of BPH. Elevated dietary inflammatory load promotes systemic inflammation and prostate-specific immune activation, potentially mediated through gut-derived inflammatory signaling. Conversely, anti-inflammatory diets rich in fiber and unsaturated fatty acids confer protective effects by attenuating inflammatory cytokine expression and preserving glandular architecture. These findings highlight the importance of diet as a modifiable determinant of prostate health and support the consideration of dietary inflammatory load as a complementary dimension in the prevention and management of BPH.

## Data Availability

The original contributions presented in the study are included in the article/Supplementary material, further inquiries can be directed to the corresponding author.

## References

[1] Chughtai B Forde JC Thomas DD Laor L Hossack T Woo HH . Benign prostatic hyperplasia. Nat Rev Dis Primers. (2016) 2:16031. doi: 10.1038/nrdp.2016.31 27147135

[2] Global incidence prevalence years lived with disability (YLDs) disability-adjusted life-years (DALYs) healthy life expectancy (HALE) for 371 diseases injuries in 204 countries . Lancet. (2024) 403:2133–61. doi: 10.1016/S0140-6736(24)00757-8 38642570 PMC11122111

[3] Wei H Zhu C Huang Q Yang J Li YT Zhang YG . Global, regional, and national burden of benign prostatic hyperplasia from 1990 to 2021 and projection to 2035. BMC Urol. (2025) 25:34. doi: 10.1186/s12894-025-01715-9 39972318 PMC11837592

[4] Wei JT Calhoun E Jacobsen SJ. Urologic diseases in America project: benign prostatic hyperplasia. J Urol. (2005) 173:1256–61. doi: 10.1097/01.ju.0000155709.37840.fe 15758764

[5] Wang Z Yuan C Zhang Y Abdelaty NS Chen C Shen J . Food inflammation index reveals the key inflammatory components in foods and heterogeneity within food groups: how do we choose food? J Adv Res. (2025) 74:87–98. doi: 10.1016/j.jare.2024.10.010 39401693 PMC12302825

[6] Baechle JJ Chen N Makhijani P Winer S Furman D Winer DA. Chronic inflammation and the hallmarks of aging. Mol Metab. (2023) 74:101755. doi: 10.1016/j.molmet.2023.101755 37329949 PMC10359950

[7] Liu Z Liang Q Ren Y Guo C Ge X Wang L . Immunosenescence: molecular mechanisms and diseases. Signal Transduct Target Ther. (2023) 8:200. doi: 10.1038/s41392-023-01451-2 37179335 PMC10182360

[8] Snelson M Tan SM Clarke RE de Pasquale C Thallas-Bonke V Nguyen TV . Processed foods drive intestinal barrier permeability and microvascular diseases. Sci Adv. (2021) 7:eabe4841. doi: 10.1126/sciadv.abe4841 33789895 PMC8011970

[9] Tallima H El Ridi R. Arachidonic acid: physiological roles and potential health benefits - a review. J Adv Res. (2018) 11:33–41. doi: 10.1016/j.jare.2017.11.004 30034874 PMC6052655

[10] Thorburn AN Macia L Mackay CR. Diet, metabolites, and “western-lifestyle” inflammatory diseases. Immunity. (2014) 40:833–42. doi: 10.1016/j.immuni.2014.05.014 24950203

[11] ElJalby M Thomas D Elterman D Chughtai B. The effect of diet on BPH, LUTS, and ED. World J Urol. (2019) 37:1001–5. doi: 10.1007/s00345-018-2568-0 30470872

[12] Dagli I Uzel T Canbolat MZ Demirci A Hizli F. The mediterranean diet and benign prostatic hyperplasia: a pathway to improved urinary health. Prostate. (2025) 85:1222–6. doi: 10.1002/pros.70009 40619693

[13] Lopez-Garcia E Schulze MB Fung TT Meigs JB Rifai N Manson JE . Major dietary patterns are related to plasma concentrations of markers of inflammation and endothelial dysfunction. Am J Clin Nutr. (2004) 80:1029–35. doi: 10.1093/ajcn/80.4.1029 15447916

[14] Phillips CM Chen LW Heude B Bernard JY Harvey NC Duijts L . Dietary inflammatory index and non-communicable disease risk: a narrative review. Nutrients. (2019) 11:1873. doi: 10.3390/nu11081873 31408965 PMC6722630

[15] Jiang S Liu H Li C. Dietary regulation of oxidative stress in chronic metabolic diseases. Foods. (2021) 10:1854. doi: 10.3390/foods10081854 34441631 PMC8391153

[16] Martínez-González MA Salas-Salvadó J Estruch R Corella D Fitó M Ros E. Benefits of the mediterranean diet: insights from the PREDIMED study. Prog Cardiovasc Dis. (2015) 58:50–60. doi: 10.1016/j.pcad.2015.04.003 25940230

[17] Hill JO Wyatt HR Peters JC. Energy balance and obesity. Circulation. (2012) 126:126–32. doi: 10.1161/CIRCULATIONAHA.111.087213 22753534 PMC3401553

[18] Minihane AM Vinoy S Russell WR Baka A Roche HM Tuohy KM . Low-grade inflammation, diet composition and health: current research evidence and its translation. Br J Nutr. (2015) 114:999–1012. doi: 10.1017/S0007114515002093 26228057 PMC4579563

[19] Khan S Wirth MD Ortaglia A Alvarado CR Shivappa N Hurley TG . Design, development and construct validation of the children's dietary inflammatory index. Nutrients. (2018) 10:993. doi: 10.3390/nu10080993 30061487 PMC6115957

[20] Shivappa N Steck SE Hurley TG Hussey JR Hébert JR. Designing and developing a literature-derived, population-based dietary inflammatory index. Public Health Nutr. (2014) 17:1689–96. doi: 10.1017/S1368980013002115 23941862 PMC3925198

[21] Devjani S Vedula P Javadi SS Smith B Han G Wu JJ. Association of people with atopic dermatitis and household income among US adults in the 1999-2006 national health and nutrition examination survey. J Eur Acad Dermatol Venereol. (2023) 37:e1112–e4. doi: 10.1111/jdv.19140 37113035

[22] Hébert JR Shivappa N Wirth MD Hussey JR Hurley TG. Perspective: The dietary inflammatory index (DII)-lessons learned, improvements made, and future directions. Adv Nutr. (2019) 10:185–95. doi: 10.1093/advances/nmy071 30615051 PMC6416047

[23] Ahluwalia N Dwyer J Terry A Moshfegh A Johnson C. Update on NHANES dietary data: focus on collection, release, analytical considerations, and uses to inform public policy. Adv Nutr. (2016) 7:121–34. doi: 10.3945/an.115.009258 26773020 PMC4717880

[24] Wang X Hu J Liu L Zhang Y Dang K Cheng L . Association of dietary inflammatory index and dietary oxidative balance score with all-cause and disease-specific mortality: findings of 2003-2014 national health and nutrition examination survey. Nutrients. (2023) 15:3148. doi: 10.3390/nu15143148 37513566 PMC10383761

[25] Shivappa N Hebert JR Marcos A Diaz LE Gomez S Nova E . Association between dietary inflammatory index and inflammatory markers in the HELENA study. Mol Nutr Food Res. (2017) 61:10.1002/mnfr.201600707. doi: 10.1002/mnfr.201600707 27981781 PMC5517083

[26] Wirth MD Shivappa N Davis L Hurley TG Ortaglia A Drayton R . Construct validation of the dietary inflammatory index among African Americans. J Nutr Health Aging. (2017) 21:487–91. doi: 10.1007/s12603-016-0775-1 28448077 PMC5547883

[27] Feng X Chen Y Xia W Zhang B. Association between dietary niacin intake and benign prostatic hyperplasia: a population-based results from NHANES 2003-2008. J Health Popul Nutr. (2024) 43:130. doi: 10.1186/s41043-024-00624-1 39174993 PMC11342560

[28] Zhou H Xu M Hao X Xu Z Pan Y Liu X. Association of serum uric acid levels with benign prostatic hyperplasia in US men: results from NHANES 2005-2008. Aging Male. (2023) 26:2275775. doi: 10.1080/13685538.2023.2275775 37897234

[29] Zhou H Xu M Pan Y Wang S Xu Z Liu L . The association between several serum micronutrients and benign prostatic hyperplasia: results from NHANES 2003-2006. Prostate. (2024) 84:212–20. doi: 10.1002/pros.24641 37899678

[30] Wang X Yu Q Michel MC. Editorial: benign prostatic hyperplasia and overactive bladder: new members of metabolic syndrome. Front Urol. (2023) 3:1272592. doi: 10.3389/fruro.2023.1272592 40778039 PMC12327302

[31] Burgess S Butterworth A Thompson SG. Mendelian randomization analysis with multiple genetic variants using summarized data. Genet Epidemiol. (2013) 37:658–65. doi: 10.1002/gepi.21758 24114802 PMC4377079

[32] Bowden J Davey Smith G Haycock PC Burgess S. Consistent estimation in Mendelian randomization with some invalid instruments using a weighted median estimator. Genet Epidemiol. (2016) 40:304–14. doi: 10.1002/gepi.21965 27061298 PMC4849733

[33] Liu Y Lai H Zhang R Xia L Liu L. Causal relationship between gastro-esophageal reflux disease and risk of lung cancer: insights from multivariable Mendelian randomization and mediation analysis. Int J Epidemiol. (2023) 52:1435–47. doi: 10.1093/ije/dyad090 37344162

[34] Verbanck M Chen CY Neale B Do R. Publisher correction: detection of widespread horizontal pleiotropy in causal relationships inferred from Mendelian randomization between complex traits and diseases. Nat Genet. (2018) 50:1196. doi: 10.1038/s41588-018-0164-2 29967445

[35] De Nunzio C Presicce F Tubaro A. Inflammatory mediators in the development and progression of benign prostatic hyperplasia. Nat Rev Urol. (2016) 13:613–26. doi: 10.1038/nrurol.2016.168 27686153

[36] Reeves PG Nielsen FH Fahey GC Jr. AIN-93 purified diets for laboratory rodents: final report of the American Institute of Nutrition ad hoc writing committee on the reformulation of the AIN-76A rodent diet. J Nutr. (1993) 123:1939–51. doi: 10.1093/jn/123.11.1939 8229312

[37] Calder PC. Omega-3 fatty acids and inflammatory processes. Nutrients. (2010) 2:355–74. doi: 10.3390/nu2030355 22254027 PMC3257651

[38] Djuricic I Calder PC. Beneficial outcomes of omega-6 and omega-3 polyunsaturated fatty acids on human health: an update for 2021. Nutrients. (2021) 13:2421. doi: 10.3390/nu13072421 34371930 PMC8308533

[39] Zhao J Cheng W Lu H Shan A Zhang Q Sun X . High fiber diet attenuate the inflammation and adverse remodeling of myocardial infarction via modulation of gut microbiota and metabolites. Front Microbiol. (2022) 13:1046912. doi: 10.3389/fmicb.2022.1046912 36620030 PMC9810810

[40] Varghese F Bukhari AB Malhotra R De A IHC. Profiler: an open source plugin for the quantitative evaluation and automated scoring of immunohistochemistry images of human tissue samples. PLoS ONE. (2014) 9:e96801. doi: 10.1371/journal.pone.0096801 24802416 PMC4011881

[41] Bowden J Holmes MV. Meta-analysis and Mendelian randomization: a review. Res Synth Methods. (2019) 10:486–96. doi: 10.1002/jrsm.1346 30861319 PMC6973275

[42] Canto-Osorio F Denova-Gutierrez E Sánchez-Romero LM Salmerón J Barrientos-Gutierrez T. Dietary inflammatory index and metabolic syndrome in Mexican adult population. Am J Clin Nutr. (2020) 112:373–80. doi: 10.1093/ajcn/nqaa135 32511694

[43] Zhang J Jia J Lai R Wang X Chen X Tian W. et al. Association between dietary inflammatory index and atherosclerosis cardiovascular disease in US adults. Front Nutr. (2022) 9:1044329. doi: 10.3389/fnut.2022.1044329 36687707 PMC9849765

[44] Dai YN Yi-Wen Yu E Zeegers MP Wesselius A. The association between dietary inflammatory potential and urologic cancers: a meta-analysis. Adv Nutr. (2024) 15:100124. doi: 10.1016/j.advnut.2023.09.012 37940476 PMC10831898

[45] Gandaglia G Briganti A Gontero P Mondaini N Novara G Salonia A . The role of chronic prostatic inflammation in the pathogenesis and progression of benign prostatic hyperplasia (BPH). BJU Int. (2013) 112:432–41. doi: 10.1111/bju.12118 23650937

[46] Cao D Sun R Peng L Li J Huang Y Chen Z . Immune cell proinflammatory microenvironment and androgen-related metabolic regulation during benign prostatic hyperplasia in aging. Front Immunol. (2022) 13:842008. doi: 10.3389/fimmu.2022.842008 35386711 PMC8977548

[47] Sanderson E Davey Smith G Windmeijer F Bowden J. An examination of multivariable Mendelian randomization in the single-sample and two-sample summary data settings. Int J Epidemiol. (2019) 48:713–27. doi: 10.1093/ije/dyy262 30535378 PMC6734942

[48] Cao H Shi C Aihemaiti Z Dai X Wang F Wang S. Association between circulating inflammatory proteins and benign prostatic disease: a Mendelian randomization study. Sci Rep. (2024) 14:23667. doi: 10.1038/s41598-024-74737-2 39390078 PMC11467427

[49] Siqueira MHB. Risk factors for benign prostatic hyperplasia: a comprehensive review. Rev Assoc Med Bras. (2025) 71:e20250343. doi: 10.1590/1806-9282.20250343 40638483 PMC12245072

[50] Tristan Asensi M Napoletano A Sofi F Dinu M. Low-grade inflammation and ultra-processed foods consumption: a review. Nutrients. (2023) 15:1546. doi: 10.3390/nu15061546 36986276 PMC10058108

[51] Guo Q Jin Y Chen X Ye X Shen X Lin M . NF-κB in biology and targeted therapy: new insights and translational implications. Signal Transduct Target Ther. (2024) 9:53. doi: 10.1038/s41392-024-01757-9 38433280 PMC10910037

[52] Hu X Li J Fu M Zhao X Wang W. The JAK/STAT signaling pathway: from bench to clinic. Signal Transduct Target Ther. (2021) 6:402. doi: 10.1038/s41392-021-00791-1 34824210 PMC8617206

[53] Khan A Alzahrani HA Felemban SG Algarni AS Alenezi ABS Kamal M . Exploring TGF-β signaling in benign prostatic hyperplasia: from cellular senescence to fibrosis and therapeutic implications. Biogerontology. (2025) 26:79. doi: 10.1007/s10522-025-10226-x 40159577

[54] Gao Y Liu P He F Yang X Wu R Chen W . Fibroblast growth factor 2 promotes bladder hypertrophy caused by partial bladder outlet obstruction. Front Cell Dev Biol. (2021) 9:630228. doi: 10.3389/fcell.2021.630228 33859983 PMC8042216

[55] Silva SA Gobbo MG Pinto-Fochi ME Rafacho A Taboga SR Almeida EA . Prostate hyperplasia caused by long-term obesity is characterized by high deposition of extracellular matrix and increased content of MMP-9 and VEGF. Int J Exp Pathol. (2015) 96:21–30. doi: 10.1111/iep.12107 25529509 PMC4352349

[56] Yu X Pu H Voss M. Overview of anti-inflammatory diets and their promising effects on non-communicable diseases. Br J Nutr. (2024) 132:898–918. doi: 10.1017/S0007114524001405 39411832 PMC11576095

[57] Sato H Narita S Ishida M Takahashi Y Mingguo H Kashima S . Specific gut microbial environment in lard diet-induced prostate cancer development and progression. Int J Mol Sci. (2022) 23:2214. doi: 10.3390/ijms23042214 35216332 PMC8878430

[58] Chen J Chen B Lin B Huang Y Li J Li J . The role of gut microbiota in prostate inflammation and benign prostatic hyperplasia and its therapeutic implications. Heliyon. (2024) 10:e38302. doi: 10.1016/j.heliyon.2024.e38302 39386817 PMC11462338

[59] Mostafavi Abdolmaleky H Zhou JR. Gut microbiota dysbiosis, oxidative stress, inflammation, and epigenetic alterations in metabolic diseases. Antioxidants. (2024) 13:985. doi: 10.3390/antiox13080985 39199231 PMC11351922

[60] Zheng J Hoffman KL Chen JS Shivappa N Sood A Browman GJ . Dietary inflammatory potential in relation to the gut microbiome: results from a cross-sectional study. Br J Nutr. (2020) 124:931–42. doi: 10.1017/S0007114520001853 32475373 PMC7554089

[61] Trakman GL Fehily S Basnayake C Hamilton AL Russell E. Wilson-O'Brien A, et al. Diet and gut microbiome in gastrointestinal disease. J Gastroenterol Hepatol. (2022) 37:237–45. doi: 10.1111/jgh.15728 34716949

[62] Faqerah N Walker D Gerasimidis K. Review article: the complex interplay between diet and escherichia coli in inflammatory bowel disease. Aliment Pharmacol Ther. (2023) 58:984–1004. doi: 10.1111/apt.17720 37771255

[63] Jain S Dash P Minz AP Satpathi S Samal AG Behera PK . Lipopolysaccharide (LPS) enhances prostate cancer metastasis potentially through NF-κB activation and recurrent dexamethasone administration fails to suppress it *in vivo*. Prostate. (2019) 79:168–82. doi: 10.1002/pros.23722 30264470

[64] Wang GC Huang TR Wang KY Wu ZL Xie JB Zhang HL . Inflammation induced by lipopolysaccharide advanced androgen receptor expression and epithelial-mesenchymal transition progress in prostatitis and prostate cancer. Transl Androl Urol. (2021) 10:4275–87. doi: 10.21037/tau-21-964 34984192 PMC8661260

[65] Li Q von Ehrlich-Treuenstätt V Schardey J Wirth U Zimmermann P Andrassy J . Gut barrier dysfunction and bacterial lipopolysaccharides in colorectal cancer. J Gastrointest Surg. (2023) 27:1466–72. doi: 10.1007/s11605-023-05654-4 36973501 PMC10366024

[66] Zheng Z Wang B. The gut-liver axis in health and disease: the role of gut microbiota-derived signals in liver injury and regeneration. Front Immunol. (2021) 12:775526. doi: 10.3389/fimmu.2021.775526 34956204 PMC8703161

[67] Cani PD Amar J Iglesias MA Poggi M Knauf C Bastelica D . Metabolic endotoxemia initiates obesity and insulin resistance. Diabetes. (2007) 56:1761–72. doi: 10.2337/db06-1491 17456850

[68] Colldén H Landin A Wallenius V Elebring E Fändriks L Nilsson ME . The gut microbiota is a major regulator of androgen metabolism in intestinal contents. Am J Physiol Endocrinol Metab. (2019) 317:E1182–92. doi: 10.1152/ajpendo.00338.2019 31689143 PMC6962501

[69] Arp G Jiang AK Dufault-Thompson K Levy S Zhong A Wassan JT . Identification of gut bacteria reductases that biotransform steroid hormones. Nat Commun. (2025) 16:6285. doi: 10.1038/s41467-025-61425-6 40628728 PMC12238238

[70] Liu XF Shao JH Liao YT Wang LN Jia Y Dong PJ . Regulation of short-chain fatty acids in the immune system. Front Immunol. (2023) 14:1186892. doi: 10.3389/fimmu.2023.1186892 37215145 PMC10196242

[71] Sultan MI Ibrahim SA Youssef RF. Impact of a mediterranean diet on prevention and management of urologic diseases. BMC Urol. (2024) 24:48. doi: 10.1186/s12894-024-01432-9 38408996 PMC10898175

[72] Al-Juhani A Desoky MS Almuhaimid AA Zaheer M Alhaqbani HF Abalkhail EA . Efficacy of gut microbiome-targeted therapies in modulating systemic inflammation and low-grade chronic inflammatory states in adults with metabolic disorders: a systematic review. Cureus. (2025) 17:e92881. doi: 10.7759/cureus.92881 41133047 PMC12541121

[73] Ceapa C Wopereis H Rezaïki L Kleerebezem M Knol J Oozeer R. Influence of fermented milk products, prebiotics and probiotics on microbiota composition and health. Best Pract Res Clin Gastroenterol. (2013) 27:139–55. doi: 10.1016/j.bpg.2013.04.004 23768559

