# Supplementary material for: The impact of dietary inflammation index on benign prostatic hyperplasia: insights from patient data and animal models
Source: Front Nutr. 2026 Feb 24;13:1760675. doi: 10.3389/fnut.2026.1760675 (PMC12973186; doi:10.3389/fnut.2026.1760675)
Supplement: Supplementary file 2 [file Supplementary_file_2.docx]

**Supplementary Table 4. ELISA assay raw absorbance value and standard curve**

1.1 Standard Curve for TNF-α

| Raw OD450 values | Blank-corrected OD450 values | Standard Concentration (pg/ml) |
| --- | --- | --- |
| 1.48248 | 1.4412 | 1000 |
| 1.01498 | 0.9737 | 500 |
| 0.68708 | 0.6458 | 250 |
| 0.45908 | 0.4178 | 125 |
| 0.31018 | 0.2689 | 65 |
| 0.20328 | 0.1620 | 32.5 |
| 0.04128 | 0.0000 | 0 |

Standard curve for ELISA assays of TNF-α was generated using serial dilutions of recombinant standards. Absorbance was measured at 450 nm, and concentrations were calculated using four-parameter logistic regression.

1.2 TNF-α Concentration (pg/ml)

| Group | Animal ID | Raw OD450 values | Blank-corrected OD450 values | TNF-α Concentration(pg/ml) |
| --- | --- | --- | --- | --- |
| Control | C1 | 0.4489 | 0.4076 | 120.34 |
| Control | C2 | 0.5318 | 0.4906 | 160.52 |
| Control | C3 | 0.4588 | 0.4175 | 124.87 |
| Control | C4 | 0.5247 | 0.4834 | 156.83 |
| Control | C5 | 0.4580 | 0.4168 | 124.51 |
| Control | C6 | 0.5220 | 0.4808 | 155.49 |
| Control | C7 | 0.4913 | 0.4500 | 140.22 |
| Control | C8 | 0.4961 | 0.4548 | 142.55 |
| Pro-inflammatory | P1 | 0.5033 | 0.4620 | 146.09 |
| Pro-inflammatory | P2 | 0.5509 | 0.5096 | 170.48 |
| Pro-inflammatory | P3 | 0.5951 | 0.5538 | 194.74 |
| Pro-inflammatory | P4 | 0.6368 | 0.5955 | 218.97 |
| Pro-inflammatory | P5 | 0.5287 | 0.4875 | 158.92 |
| Pro-inflammatory | P6 | 0.6154 | 0.5741 | 206.33 |
| Pro-inflammatory | P7 | 0.5732 | 0.5320 | 182.56 |
| Pro-inflammatory | P8 | 0.5788 | 0.5375 | 185.61 |
| Anti-inflammatory | A1 | 0.4503 | 0.4090 | 120.96 |
| Anti-inflammatory | A2 | 0.4409 | 0.3996 | 116.74 |
| Anti-inflammatory | A3 | 0.4679 | 0.4266 | 129.07 |
| Anti-inflammatory | A4 | 0.4736 | 0.4323 | 131.75 |
| Anti-inflammatory | A5 | 0.4785 | 0.4372 | 134.08 |
| Anti-inflammatory | A6 | 0.4289 | 0.3876 | 111.43 |
| Anti-inflammatory | A7 | 0.4780 | 0.4368 | 133.85 |
| Anti-inflammatory | A8 | 0.4632 | 0.4219 | 126.9 |

2.1 Standard Curve for IL-1β

| Raw OD450 values | Blank-corrected OD450 values | Standard Concentration (pg/ml) |
| --- | --- | --- |
| 1.6734 | 1.6350 | 1000 |
| 1.1309 | 1.0925 | 500 |
| 0.7645 | 0.7261 | 250 |
| 0.4876 | 0.4492 | 125 |
| 0.3275 | 0.2891 | 62.5 |
| 0.2001 | 0.1617 | 31.2 |
| 0.1313 | 0.0929 | 15.6 |
| 0.0384 | 0.0000 | 0 |

Standard curve for ELISA assays of IL-1β was generated using serial dilutions of recombinant standards. Absorbance was measured at 450 nm, and concentrations were calculated using four-parameter logistic regression.

2.2 IL-1β Concentration (pg/ml)

| Group | Animal ID | Raw OD450 values | Blank-corrected OD450 values | IL -1β Concentration(pg/ml) |
| --- | --- | --- | --- | --- |
| Control | C1 | 0.6005 | 0.5621 | 170.11 |
| Control | C2 | 0.6405 | 0.6021 | 189.15 |
| Control | C3 | 0.6578 | 0.6194 | 197.67 |
| Control | C4 | 0.7032 | 0.6648 | 220.87 |
| Control | C5 | 0.6533 | 0.6149 | 195.43 |
| Control | C6 | 0.5955 | 0.5571 | 167.79 |
| Control | C7 | 0.6449 | 0.6065 | 191.32 |
| Control | C8 | 0.6870 | 0.6486 | 212.47 |
| Pro-inflammatory | P1 | 0.7693 | 0.7309 | 256.76 |
| Pro-inflammatory | P2 | 0.7898 | 0.7514 | 268.38 |
| Pro-inflammatory | P3 | 0.8061 | 0.7677 | 277.84 |
| Pro-inflammatory | P4 | 0.8217 | 0.7833 | 286.97 |
| Pro-inflammatory | P5 | 0.7385 | 0.7001 | 239.73 |
| Pro-inflammatory | P6 | 0.7785 | 0.7401 | 261.91 |
| Pro-inflammatory | P7 | 0.7421 | 0.7037 | 241.65 |
| Pro-inflammatory | P8 | 0.8082 | 0.7698 | 279.03 |
| Anti-inflammatory | A1 | 0.5737 | 0.5353 | 157.87 |
| Anti-inflammatory | A2 | 0.6212 | 0.5828 | 179.85 |
| Anti-inflammatory | A3 | 0.5562 | 0.5178 | 150.09 |
| Anti-inflammatory | A4 | 0.6142 | 0.5758 | 176.53 |
| Anti-inflammatory | A5 | 0.6265 | 0.5881 | 182.37 |
| Anti-inflammatory | A6 | 0.5711 | 0.5327 | 156.72 |
| Anti-inflammatory | A7 | 0.5959 | 0.5575 | 167.97 |
| Anti-inflammatory | A8 | 0.6035 | 0.5651 | 171.52 |

3.1 Standard Curve for IL-6

| Raw OD450 values | Blank-corrected OD450 values | Standard Concentration (pg/ml) |
| --- | --- | --- |
| 1.6732 | 1.6310 | 5000 |
| 0.9705 | 0.9283 | 2500 |
| 0.5807 | 0.5385 | 1250 |
| 0.295 | 0.2528 | 625 |
| 0.176 | 0.1338 | 312.5 |
| 0.1195 | 0.0773 | 156.25 |
| 0.0865 | 0.0443 | 78.13 |
| 0.0422 | 0.0000 | 0 |

Standard curve for ELISA assays of IL-6 was generated using serial dilutions of recombinant standards. Absorbance was measured at 450 nm, and concentrations were calculated using four-parameter logistic regression.

3.2 IL-6 Concentration (pg/ml)

| Group | Animal ID | Raw OD450 values | Blank-corrected OD450 values | IL -6  Concentration(pg/ml) |
| --- | --- | --- | --- | --- |
| Control | C1 | 0.0930 | 0.0508 | 109.86 |
| Control | C2 | 0.0884 | 0.0462 | 99.74 |
| Control | C3 | 0.0838 | 0.0416 | 89.67 |
| Control | C4 | 0.0862 | 0.0440 | 95.06 |
| Control | C5 | 0.0934 | 0.0512 | 110.75 |
| Control | C6 | 0.0910 | 0.0488 | 105.49 |
| Control | C7 | 0.0920 | 0.0498 | 107.64 |
| Control | C8 | 0.0870 | 0.0448 | 96.82 |
| Pro-inflammatory | P1 | 0.1085 | 0.0683 | 148.22 |
| Pro-inflammatory | P2 | 0.1112 | 0.0690 | 149.83 |
| Pro-inflammatory | P3 | 0.1184 | 0.0762 | 165.74 |
| Pro-inflammatory | P4 | 0.1139 | 0.0717 | 155.84 |
| Pro-inflammatory | P5 | 0.1067 | 0.0645 | 139.76 |
| Pro-inflammatory | P6 | 0.1099 | 0.0677 | 146.98 |
| Pro-inflammatory | P7 | 0.1115 | 0.0693 | 150.52 |
| Pro-inflammatory | P8 | 0.1110 | 0.0688 | 149.33 |
| Anti-inflammatory | A1 | 0.0770 | 0.0348 | 75.08 |
| Anti-inflammatory | A2 | 0.0758 | 0.0336 | 72.33 |
| Anti-inflammatory | A3 | 0.0816 | 0.0394 | 85.02 |
| Anti-inflammatory | A4 | 0.0845 | 0.0423 | 91.28 |
| Anti-inflammatory | A5 | 0.0790 | 0.0368 | 79.43 |
| Anti-inflammatory | A6 | 0.0816 | 0.0394 | 85.01 |
| Anti-inflammatory | A7 | 0.0849 | 0.0427 | 92.07 |
| Anti-inflammatory | A8 | 0.0832 | 0.0410 | 88.51 |
